# Opportunities to Improve Resilience in Animal Breeding Programs

**DOI:** 10.3389/fgene.2018.00692

**Published:** 2019-01-14

**Authors:** Tom V. L. Berghof, Marieke Poppe, Han A. Mulder

**Affiliations:** Wageningen University & Research Animal Breeding and Genomics, Wageningen, Netherlands

**Keywords:** resilience, livestock, breeding program, micro-environment, macro-environment, economic value, big data, longitudinal data

## Abstract

Resilience is the capacity of an animal to be minimally affected by disturbances or to rapidly return to the state pertained before exposure to a disturbance. However, indicators for general resilience to environmental disturbances have not yet been defined, and perhaps therefore resilience is not yet included in breeding goals. The current developments on big data collection give opportunities to determine new resilience indicators based on longitudinal data, which can aid to incorporate resilience in animal breeding goals. The objectives of this paper were: (1) to define resilience indicator traits based on big data, (2) to define economic values for resilience, and (3) to show the potential to improve resilience of livestock through inclusion of resilience in breeding goals. Resilience might be measured based on deviations from expected production levels over a period of time. Suitable resilience indicators could be the variance of deviations, the autocorrelation of deviations, the skewness of deviations, and the slope of a reaction norm. These (new) resilience indicators provide opportunity to include resilience in breeding programs. Economic values of resilience indicators in the selection index can be calculated based on reduced costs due to labor and treatments. For example, when labor time is restricted, the economic value of resilience increases with an increasing number of animals per farm, and can become as large as the economic value of production. This shows the importance of including resilience in breeding goals. Two scenarios were described to show the additional benefit of including resilience in breeding programs. These examples showed that it is hard to improve resilience with only production traits in the selection index, but that it is possible to greatly improve resilience by including resilience indicators in the selection index. However, when health-related traits are already present in the selection index, the effect is smaller. Nevertheless, inclusion of resilience indicators in the selection index increases the response in the breeding goal and resilience, which results in less labor-demanding, and thus easier-to-manage livestock. Current developments on massive collection of data, and new phenotypes based on these data, offer exciting opportunities to breed for improved resilience of livestock.

## Introduction

Modern livestock production is characterized by intensification, i.e., a higher number of animals per farm. To achieve successful intensification of livestock production, without negative effects on animals, farmers and farms, certain requirements need to be met. One of these requirements for intensification of livestock production is the capability of the farmer to take care of a larger number of animals. This requires healthy and easy-to-manage animals that need little/less attention time (Elgersma et al., 2018). Resilient animals are animals that need little/less attention time: increasing resilience is therefore desired. Improvement of resilience can be realized by different strategies. One strategy is to increase resilience by genetic selection in breeding programs. The advantage of genetic selection, in contrast to management improvements, is that it is cumulative and affects all subsequent generations of livestock.

We define resilience as “the capacity of the animal to be minimally affected by disturbances or to rapidly return to the state pertained before exposure to a disturbance” (adjusted from Colditz and Hine, 2016). Several definitions of resilience (e.g., Colditz and Hine, 2016), and resilience-associated concepts have been discussed in literature: robustness, tolerance, resistance, GxE interaction, genetic heterogeneity of environmental variance, plasticity, environmental sensitivity, canalization, (developmental) stability, and residual within-individual phenotypic variation (e.g., Holling, 1973; Debat and David, 2001; De Jong and Bijma, 2002; Flatt, 2005; Knap, 2005; Mulder et al., 2007, 2013; Bishop, 2012; Westneat et al., 2015; Colditz and Hine, 2016; Marjanovic et al., 2018). We have summarized these definitions and concepts in Box 1, but it is beyond the scope of this paper to discuss differences and similarities between these.

Box 1 **Definitions of resilience and resilience-associated concepts discussed in literature (quoted). Note that this box does not serve as a critical reflection of literature, but as an overview for interpretation of different concepts.**

**Resilience**: the capacity of the animal to be minimally affected by disturbances or to rapidly return to the state pertained before exposure to a disturbance (adjusted from Colditz and Hine, 2016; this paper).

**Robustness**: animals that combine high production potential with resilience to external stressors, allowing for unproblematic expression of high production potential in a wide variety of environmental conditions (adjusted from Knap, 2005).

**Tolerance**: the net impact on performance of a given level of disturbance (adjusted from Bishop, 2012).

**Resistance**: the ability of the host animal to exert control over the disturbance (adjusted from Bishop, 2012).

**GxE interaction**: the best genotype in one environment may not be the best genotype in another environment and those genotypes differ in their response to environmental factors (Mulder et al., 2013).

**Environmental sensitivity**: loss of flexibility to deal with intensive or limiting conditions, due to unbalanced resource allocation (Knap, 2005).

**Heritable variation in environmental variance** or **inherited variability**: the variability of trait values of a genotype, measured either repeatedly on the same individual, or on multiple individuals belonging to the same family (Marjanovic et al., 2018).

**(Phenotypic) plasticity**: the ability of an organism to alter its physiology, morphology or development in response to changes in its environment (Debat and David, 2001).

**Macro-environmental sensitivity:** the property of the organisms of a genotype to develop systematically different phenotypes in different environments = norm of reaction (Debat and David, 2001).

**(Genetic) canalization**: similarity of the expression of the phenotypic character under different conditions of development; the process by which phenotypic variation is reduced by developmental mechanisms or a genotype's phenotype remains relatively invariant when individuals of the same single- or multilocus genotype differ in their genetic background; a particular kind of epistasis (Debat and David, 2001; Flatt, 2005).

**(Developmental) stability**: the ability of a system to return to an equilibrium state after a temporary disturbance (Holling, 1973).

**Residual within-individual phenotypic variation**: amount of within-individual variance not explained in a specific statistical model (i.e., the average squared deviations of observations from an individual's reaction norm), averaged over a sample of individuals (Westneat et al., 2015).

Disturbances can be of different nature, being either physical (e.g., disease, temperature stress) or psychological (e.g., novel environment, social stressor, human interaction) (see Colditz and Hine, 2016 for a review). “General” resilience is therefore a composite trait, consisting of different resilience types depending on the nature of the disturbance (Colditz and Hine, 2016; Elgersma et al., 2018). Disturbances can be categorized as “macro-environmental factors” or “micro-environmental factors” (Falconer and Mackay, 1996; Mulder et al., 2013). Macro-environmental factors are characteristics of an environment and thus affect the (majority of the) whole population within that macro-environment (e.g., disease pressure, ambient temperature). Genetic variation in the response to these macro-environmental factors can be expressed as the genetic variance of the slope of a reaction norm over different environments or different quantities of a disturbance (Mulder et al., 2013). Micro-environmental factors occur within a macro-environment, and thus affect only a minority of the whole population within that macro-environment (e.g., diseases, social interactions). Genetic differences in response to micro-environmental factors can be expressed as genetic variance in the size of environmental variance (Mulder et al., 2013). Despite the fact that macro- and micro-environmental sensitivities refer to different concepts and are best investigated with different methods (Mulder et al., 2015a), the estimated genetic correlation between them was high (0.76 with SE = 0.10; Mulder et al., 2013). This indicates that, even though disturbances have effects on a different scale, resilience to these disturbances have a common genetic background and will respond to selection for increased resilience in a similar direction. Nevertheless, from a practical point of view, resilience to occasional macro-environmental disturbances, such as disease outbreaks and heat waves, are less frequent and therefore of lesser importance, at least in most farms in temperate climates. Thus easy-to-manage livestock is livestock with increased resilience to day-to-day micro-environmental disturbances within the macro-environment (i.e., a farm), but also with increased resilience to macro-environmental disturbances.

Resilience indicators are not yet included in selection indices as far as we know, despite their clear relevance for healthy and easy-to-manage livestock (and also production uniformity and production efficiency). This is likely due to ignorance on how to define, measure and weight resilience indicators and their economic values, and due to the belief that current health-related traits in selection indices (e.g., longevity, mortality, growth) cover resilience, at least mostly (e.g., Knap, 2005). Although suggestions for measuring environmental sensitivity (Knap, 2009b) (i.e., resilience) and determining its economic value have been proposed (Knap, 2005), proper data collection and data processing tools were considered to be major challenges for implementation (Knap, 2009b). However, recent technological developments allow collection of big/longitudinal data and derivation of new phenotypes from it (Mulder, 2017). These developments will only continue to expand and will become more important in the future. New ideas to explore and exploit big data and new phenotypes are required. Also, health-related traits do not necessarily reflect general resilience, but mainly capture resistance to diseases (i.e., disease resilience; Mulder and Rashidi (2017). In addition, breeding value estimation for disease-related traits is limited to only a few diseases, if any. Thus there seems to be additional benefit for including general resilience indicators in selection indices.

The objectives of this paper were therefore:

1. to define resilience indicator traits based on big/longitudinal data,
2. to define economic values for resilience indicators based on labor and treatment costs, and
3. to show the potential to improve resilience of livestock through inclusion of resilience in breeding goals.

The paper addresses the described objectives sequentially and ends with overall conclusions, and therefore the paper differs in its setup from conventional research papers.

## Defining Resilience Indicators

Resilience has been a popular concept in a broad range of scientific disciplines in the last two decades (e.g., Scheffer et al., 2015; Ge et al., 2016). However, defining resilience indicators has been proven to be difficult, and different options have been proposed, which suggests that the “right” resilience indicator has yet to be defined. Recent technological developments give new opportunities to explore alternative resilience indicators based on longitudinal data (Mulder, 2017). Here we propose resilience indicators, which exploit the availability of many repeated observations per individual or per (sire-)family (i.e., big data). We will also describe some of the conditions these resilience indicators will have to fulfill in order to be informative, and their potential weaknesses.

### Resilience Indicators

Measuring general resilience is difficult. Many studies have focused on one particular type of resilience (especially disease resilience) and have used experimental set-ups to identify underlying physiological mechanisms. However, these mechanisms strongly depend on the nature of the disturbance, are often chosen based on the interest of the study, and might characterize a phenotype that is (too) dependent on the investigated disturbance (Colditz and Hine, 2016). Even though these studies can provide useful information and insights in physiology, results may not be representative of general resilience. Furthermore, typically these challenge environments deviate from commercial environments and might therefore be less representative due to GxE interactions. Instead it has been proposed to measure “*summary characteristics of response variables*” (quoted from Colditz and Hine, 2016) as general resilience indicators (Knap, 2009b; Doeschl-Wilson et al., 2012; Colditz and Hine, 2016; Elgersma et al., 2018).

Colditz and Hine (2016) proposed a diverse set of “*summary characteristics of response variables*” to measure resilience to a disturbance, including typical production traits like feed intake, growth rate, and other production variables. Individuals experiencing a disturbance eat and produce less than (i.e., they deviate from) their potential without a disturbance (e.g., Van der Waaij et al., 2000). However, a deviation between observed production mean and estimated potential (as proposed by Colditz and Hine, 2016) does not necessarily fully cover the definition of resilience: resilient animals might have a severe drop in production, but might also have the capacity to rapidly return to the state pertained before exposure to a disturbance compared to less resilient animals. Deviations over a period of time (for at least the length of the disturbance) likely reflect resilience better. Thus, resilience might be measured based on deviations of expected production and observed production (i.e., residuals) over a period of time.

Many potential resilience indicator traits have been described, aimed at predicting changes in states of ecosystems (see Table 1 of Scheffer et al., 2015 for an elaborate overview). However, many of these resilience indicators are difficult to apply to livestock species or genetic improvement of livestock, because the resilience indicators are not suitable in livestock production data (e.g., spatial correlation, spectral reddening). In addition, a large number of investigated resilience indicators are alike, because they use the data in a similar way, i.e., the number of unique indicators is limited. However, we do see opportunities for some of them. In this paper, we elaborated on four resilience indicators, for which we propose they show a different perspective of resilience [see also Scheffer et al. (2018)]. Suitable resilience indicators based on a single trait (e.g., production, feed intake) in animal production might be *variance* of deviations, *autocorrelation* of deviations, *skewness* of deviations of production traits over a period of time, and the *slope* of a reaction norm (see Table 1). We assume that in general negative deviations are mostly observed for less resilient animals, but this might be opposite depending on the trait, e.g., body temperature.

- *Variance* of deviations (also known as uniformity, inherited variability, residual variance, or genetic heterogeneity of environmental variance (see Hill and Mulder, 2010; Elgersma et al., 2018 for overviews) gives an indication of the impact of disturbances with: low(er) variance for animals without disturbances or not influenced by disturbances; and high(er) variance for animals influenced by disturbances. We hypothesize that the variance of deviations captures the severity and duration (i.e., rate of recovery) of environmental perturbations an individual experiences. The contribution of severity and duration cannot be disentangled within this resilience indicator.
- (Lag-one) *autocorrelation* of deviations gives an indication of the length of the impact of disturbances with: an autocorrelation around 0 (i.e., subsequent deviations are unrelated) for animals without disturbances, not influenced by disturbances, or with a fast recovery from disturbances; an autocorrelation toward +1 (i.e., subsequent deviations are more alike) for animals influenced by disturbances and with slow(er) recovery from disturbances; and an autocorrelation toward −1 (i.e., subsequent deviations are opposite) for animals influenced by disturbances and a fast and over-compensating response to disturbances, e.g., compensatory growth. We hypothesize that the autocorrelation of deviations captures the duration (i.e., rate of recovery) of environmental perturbations an individual experiences.
- *Skewness* of deviations gives an indication of the direction of deviations with: a skewness around 0 for animals without disturbances or not influenced by disturbances; a positive skewness for animals having (mainly) positive deviations due to positive responses to environmental improvements; and a negative skewness for animals having (mainly) negative deviations due to disturbances. We hypothesize that the skewness of deviations captures the severity of environmental perturbations an individual experiences.
- *Slope* of a reaction norm gives an indication of resilience toward a macro-environmental disturbance (Finlay and Wilkinson, 1963), e.g., disease pressure (Herrero-Medrano et al., 2015) or a heat wave (Ravagnolo and Misztal, 2000). The slope of a reaction norm is estimated based on the production of an individual given the level of a disturbance with: a slope of 0 for animals not influenced by the disturbance, and a slope below 0 for animals influenced by the disturbance with steeper, negative slopes for animals that are influenced more. We hypothesize that the slope of a reaction norm captures the severity of a macro-environmental perturbations an individual experiences.

**Table 1:** Overview of possible resilience indicators based on big/longitudinal data per animal with description, interpretation and remarks.

**Indicators**	**Description**	**Interpretation**	**Remarks**
*Variance* of deviations	Indication of the impact of disturbances	Low(er) variance for animals without disturbances or not influenced by disturbances; High(er) variance for animals influenced by disturbances; Captures the severity and duration (i.e., rate of recovery) of environmental perturbations an individual experiences.	Does not discriminate between negative and positive deviations; Also known as uniformity, inherited variability, residual variance, or environmental variance.
(Lag-one) *Autocorrelation* of deviations	Indication of the length of the impact of disturbances	An autocorrelation around 0 (i.e., subsequent deviations are unrelated) for animals without disturbances, not influenced by disturbances, or with a fast recovery from disturbances; An autocorrelation toward +1 (i.e., subsequent deviations/observations are more alike) for animals influenced by disturbances and with slow(er) recovery from disturbances; An autocorrelation toward −1 (i.e., subsequent deviations are opposite) for animals influenced by disturbances and a fast and over-compensating response to disturbances, e.g., Compensatory growth; Captures the duration (i.e., rate of recovery) of environmental perturbations an individual experiences.	Does not discriminate between negative and positive deviations; Does not give an indication of the impact of deviations.
*Skewness* of deviations	Indication of the direction of disturbances	A skewness around 0 for animals without disturbances or not influenced by disturbances; A positive skewness for animals having (mainly) positive deviations due to positive responses to environmental improvements; A negative skewness for animals having (mainly) negative deviations due to disturbances; Captures the severity of environmental perturbations an individual experiences.	Might be more sensitive to outliers.
*Slope* of a reaction norm	Indication of resilience toward a macro-environmental disturbance	A slope of 0 for animals not influenced by the disturbance; A slope below 0 for animals influenced by the disturbance with steeper, negative slopes for animals more influenced; Captures the severity of a macro-environmental perturbations an individual experiences.	Detection of disturbance can only be done on farm level; Each disturbance has its own slope, i.e., multiple slopes per individual are possible; Disentanglement of two disturbances simultaneously is not possible; Micro-environmental disturbances are not captured by the slope of a reaction norm, i.e., no general resilience indication.

*We assume that in general negative deviations are more observed for less resilient animals, but this might be opposite depending on the observed trait*.

Thus, less resilient animals are expected to have a larger variance, a positive autocorrelation, a negative skewness, and a steeper slope than the population average, assuming that the disturbance is reducing the trait value. Resilient animals are expected to have a smaller variance (i.e., closer to 0), an autocorrelation and a skewness around 0, and a slope closer to zero than the population average.

Some remarks regarding the proposed resilience indicators need to be made. A general remark to be made is that scaling can cause that animals with a higher mean have a higher variance of deviations, but they may also be genetically more sensitive, less resilient compared to animals with a low production (Falconer and Mackay, 1996). Specific remarks to be made are: the underlying assumption for both variance and autocorrelation as resilience indicators is that deviations are mainly (or solely) negative, because disturbances will reduce production. However, variance and autocorrelation do not discriminate between negative and positive deviations. Therefore, animals showing production above the expected production, like compensatory production (perhaps a desired trait, part of resilience), will be characterized as not/less resilient. On the other hand, such animals are likely to have shown a severe reduction in production due to the disturbance, clearly showing lack of resistance against the disturbance. In contrast, skewness does differentiate between positive and negative deviations, but is likely to be more sensitive to outliers due to cubic terms. The autocorrelation can also be negative, meaning that an animal shows fast and over-compensating responses to disturbances, e.g., compensatory growth. Although a negative autocorrelation is preferred over a (strong) positive autocorrelation, an animal with an autocorrelation around 0 is not affected by a disturbance at all, and is therefore preferred over a negative autocorrelation. Finally, the estimation of the slope of a reaction norm requires quantification on farm level either by a known environmental covariate, e.g., temperature (Ravagnolo and Misztal, 2000; Bohmanova et al., 2007; Carabaño et al., 2017), or by an overall drop in production on a farm as a consequence of many individual drops in production on that farm (Rashidi et al., 2014; Herrero-Medrano et al., 2015). In addition, each disturbance has its own slope, and it is also almost impossible to estimate multiple slopes for multiple disturbances occurring at the same time. However, the slope of a reaction norm can also be interpreted as (a form of) general resilience, although the slope likely does not capture the response to micro-environmental disturbances, which occur at the individual animal level, e.g., endemic diseases. Thus the slope only provides information for disturbances that cause a decline in farm performance, such as heat stress or a disease outbreak (in case of e.g., heat stress). Thereby the slope provides only information on macro-environmental disturbances. This is in contrast to the other three proposed resilience indicators based on deviations in Table 1, that can be used for epidemic (at the farm level) and endemic events (at the individual level). Therefore, these indicators provide information for estimation of breeding values on all types of disturbances and thus general resilience. But perhaps the “best” resilience indicator is yet to be defined based on a combination of the different resilience indicators: a multivariate approach of resilience, for example based on cross-correlations or eigenvalues which both capture the relation(s) between different resilience indicators based on different phenotypes (e.g., Lade and Gross, 2012; Scheffer et al., 2015; Gijzel et al., 2017), or an index of a number of indicators (i.e., a selection index approach, Hazel, 1943).

Little is known about the usefulness of the proposed resilience indicators based on deviations for livestock genetics: no studies have investigated the autocorrelations of deviations and the skewness of deviations, and only a few studies have investigated the variance of deviations. Elgersma et al. (2018) investigated the raw phenotypic variance of daily milk yield over the whole milking period and its relation to health and longevity traits in dairy cows. The raw variance of milk yield was heritable (0.10; Elgersma et al., 2018). Moreover, on a genetic level, cows with a low variance in milk yield deviations had significant fewer production-related diseases [i.e., udder health, genetic correlation (r_g_) = −0.36; ketosis, r_g_ = −0.52] and a higher longevity (r_g_ = −0.30), suggesting a higher resilience (Elgersma et al., 2018). Putz et al. (2018) showed similar results for pigs kept in a “natural challenge environment”: the variance of deviations in daily feed intake and deviations in daily duration at feeder during finishing phase were positively genetically correlated to mortality and number of treatments (Putz et al., 2018), indicating that pigs with lower variance have lower mortality and need less treatments. This means that both feed intake and feed duration are indicative for health status on a genetic level, and thus shows that variation in frequently measured traits can be indicative for resilience. Also, many other studies have investigated the heritability of uniformity in production traits, which is the same as variance in deviations: reported heritabilities ranged between 0.00 and 0.15 in almost all livestock species (see Hill and Mulder, 2010 for an overview up to 2010; Neves et al., 2011; Janhunen et al., 2012; Sae-Lim et al., 2015, 2017; Mulder et al., 2016; see Elgersma et al., 2018 for more). Furthermore, the genetic coefficients of variation were generally moderate to high, which suggests a relatively large potential for genetic improvement in uniformity. These studies illustrate good potential for the use of production deviations as indicator traits for resilience.

### Conditions for Measuring Resilience Indicators

For successful use of resilience indicators in breeding programs, certain conditions apply: collection of observations should be on many animals, observations need to be collected frequently and over a longer period of time, the environment has to be challenging and diverse to estimate general resilience, and most importantly the resilience indicators have to be informative for resilience.

**Figure 1:**
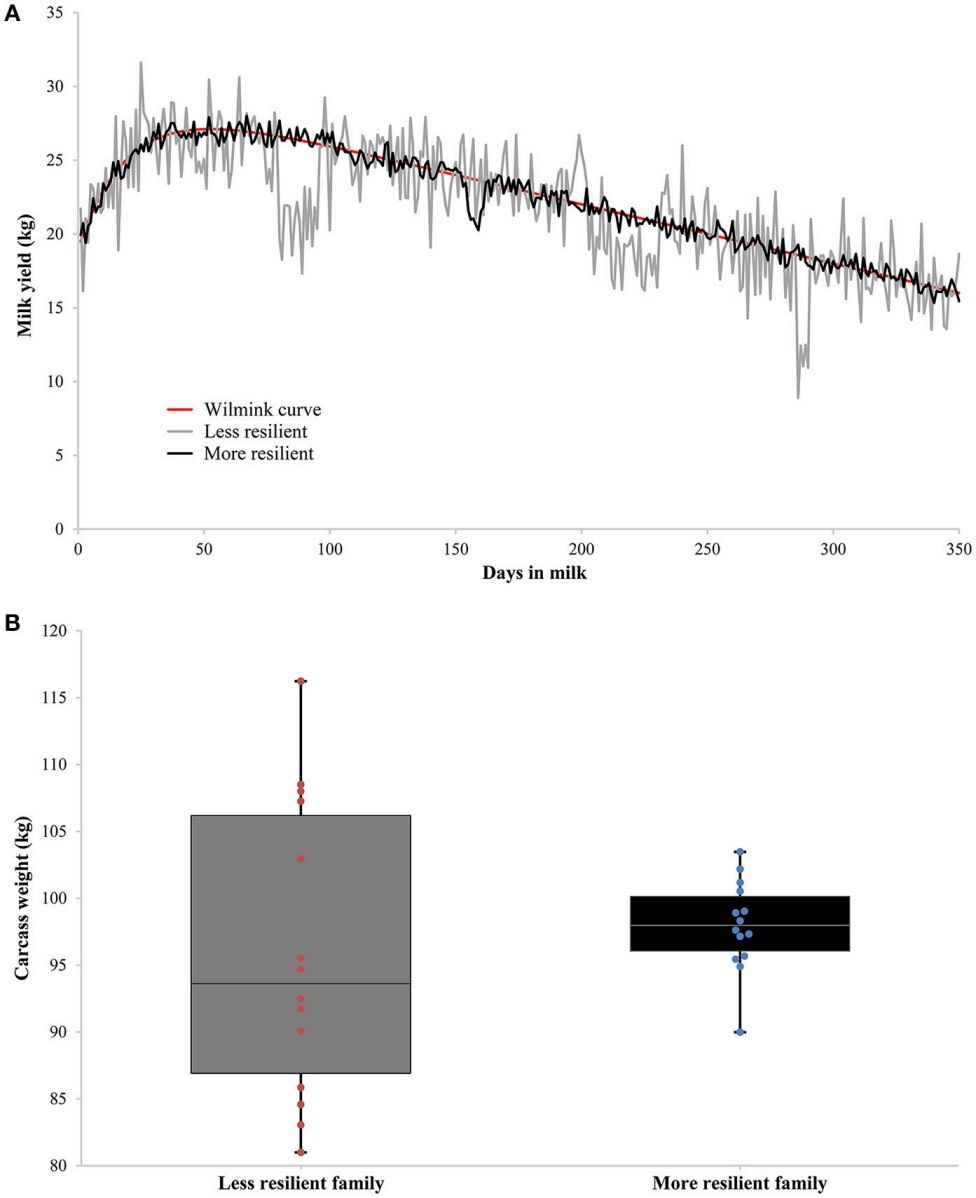
Two examples of production traits suitable for investigating resilience indicators based on deviations. **(A)** Shows repeated measurements of milk yield on individual level of two dairy cows with the same underlying Wilmink curve (in red), but differing in resilience: in gray a less resilient dairy cow with higher fluctuations in milk yield, and in black a more resilient dairy cow with lower fluctuations in milk yield. **(B)** Shows measurements of carcass weight on family level of two families differing in resilience: in gray a less resilient family with higher fluctuations in carcass weight, and in black a more resilient family with lower fluctuations in carcass weight.

Production traits suitable for investigating resilience indicators based on deviations can be either measured repeatedly on individual level or on family level (see Figure 1 for example). Repeated observations on individual level allow more accurate estimation of resilience indicators and their genetic variance and are therefore preferred. Suitable traits are for example milk yield, egg weight, body weight/growth, and feed intake. An important point that needs to be addressed is that these traits might differ in size of deviations depending on the (lactation) stage or age (for growth of young animals): standardizing deviations per stage or age might be required or treating different stages/ages as different traits. In case too few repeated observations on production traits are collected per individual, deviations of production traits collected on (sire-)family level are an alternative. Especially livestock kept in high numbers with a relatively low economic value (e.g., fish and poultry) or livestock kept in extensive farming systems (e.g., beef cattle and sheep) can benefit from such an approach to investigate resilience indicators. Suitable traits are for example slaughter/carcass traits (e.g., Ibáñez-Escriche et al., 2008) or traits recorded only during a restricted lifetime period (e.g., Neves et al., 2011; Mulder et al., 2016; Iung et al., 2017). It is to be expected that technical developments in the (near) future will allow investigation of deviations based on repeated observations on the same individual for all livestock species.

Care has to be taken to estimate deviations properly: less resilient animals will have more fluctuations in production, but as a consequence the expected production (based on own observations) will be lower. This means that deviations will be lower if simply an average expected production is used, and resilience of the animal is overestimated. For example, Elgersma et al. (2018) concluded that the raw phenotypic variance of daily milk yield is a composite measure of the residual variance and the shape of the lactation curve (Elgersma et al., 2018). They suggested to model the individual's lactation curve and use variance of deviations from the lactation curve as a better resilience indicator (Elgersma et al., 2018), as was done by Codrea et al. (2011). However, an individual permanently affected by disturbances (or a disturbance) will have a lactation curve lower than its true potential without (any) disturbances. As a consequence, deviations based on the individual's lactation curve will be lower than when compared to the individual's potential lactation curve. Modeling lactation curves based on the observed data might absorb part of the variance in deviations. Instead, deviations might be based on the difference between observed production and an individual's potential production, e.g., based on its (G)EBV. Determining the expected production of an animal is one of the major challenges for unbiased estimation of the proposed resilience indicators, and requires further investigation in the near future.

To properly estimate general resilience of individuals, two factors are essential: the length of the observation period and the frequency of the observations. First, the length of the observation period should be sufficiently long to allow different types of disturbances to occur, as was suggested by Mulder et al. (2013): in stressful environments more genetic variation for micro-environmental sensitivity can be observed compared to less stressful environments (Mulder et al., 2013). A potential risk is that results are not reproducible between or even within an environment: for instance, resilience based on disturbances caused by one particular type (e.g., diseases) does not reflect resilience toward other types of disturbance (e.g., heat), and therefore does not represent general resilience. Second, the frequency of observations should be sufficiently high to capture deviations caused by disturbances. Elgersma et al. (2018) suggested that earlier studies might have underestimated the potential of resilience indicators due to too long test-day intervals, e.g., monthly milk yield records. Also in case family deviations are used, there is a substantial risk that this does not capture any disturbance (in case no or small deviations were present), let alone a diverse set of disturbance types to estimate general resilience. In contrast, too small time periods may invoke larger noise, and it may therefore become harder to find the relevant information for resilience. The time period depends on the trait measured. Future research should focus on finding the optimal time period for determining deviations by looking at the accuracy of EBV, and the genetic correlations between the resilience indicator(s) and the existing health traits. Regardless of that, resilience is ideally estimated in an environment that has all types of disturbances for the whole production period with frequent measurements.

**Figure 2:**
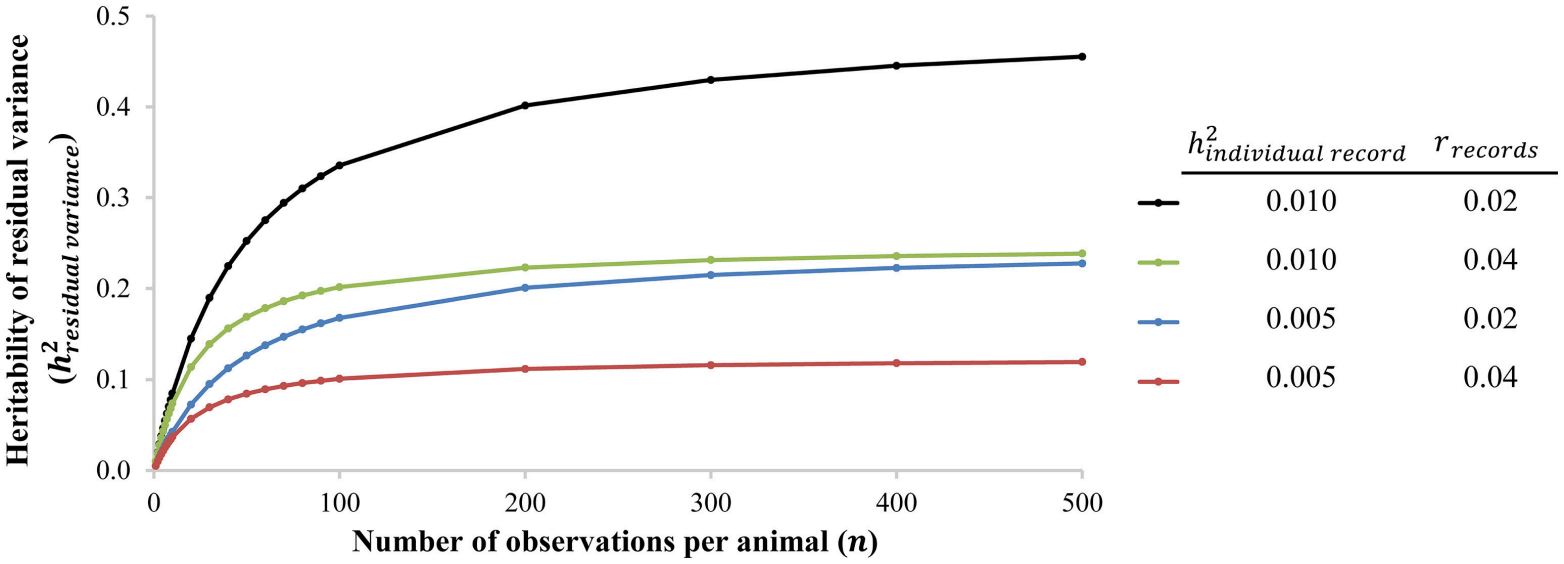
The heritability of residual variance ($h_{residual\;variance}^{2}=\frac{nh_{individual\;record}^{2}}{1+\left(n-1\right)r_{records}}$) as a function of the number of observations per animal (*n*) for two heritabilities of the residual variance based on one individual record ($h_{individual\;record}^{2}$) and two repeatabilities of records of the residual variance (*r*_*individual record*_). The used heritabilities of the residual variance based on one individual record ($h_{individual\;record}^{2}$) are similar to in-literature-reported heritabilities of residual variance based on one individual record (i.e., one single phenotypic observation), see Hill and Mulder (2010) and Elgersma et al. (2018) for examples.

Recent technological developments allow a tremendous increase in number of observations and number of observed phenotypes: big/longitudinal data and new phenotypes will result in more and new data on individuals to more accurately estimate deviations and consequently genetic parameters. Currently, many (breeding) organizations make use of routine data collection. Automatic milking systems (AMS) and automatic feeding systems (AFS) for cattle and pigs are the most well-known and well-developed examples. Other developments currently under investigation are for example automatic weighing systems and automatic recording of egg production in group housing. Collection of more data and new phenotypes are expected to increase in the near future, which will aid in (better) estimation of genetic variation of deviations: higher heritabilities are generally found with more observations (i.e., individual measurements) per individual or family (Hill and Mulder, 2010; Elgersma et al., 2018), suggesting that large datasets are required to accurately estimate genetic variance. In addition, even though heritability estimates of variance of deviations based on single records are often very low (around 0.01; see Hill and Mulder, 2010; Elgersma et al., 2018 for examples), the heritability of residual variance of many of these observations of one animal is actually moderate (Damgaard et al., 2003; Kapell et al., 2011; Sell-Kubiak et al., 2015; Mulder et al., 2016; Elgersma et al., 2018). Figure 2 shows the relationship between the heritability of the residual variance based on multiple individual records and the heritability of the residual variance based on one individual record. In summary, recent technological developments, such as AMS and AFS, allow or will allow collection of large datasets with more observations per animal (i.e., big data). This will greatly facilitate the use of resilience indicators in breeding programs of all livestock species.

## The Importance of Resilience in the Breeding Goal

Determining the breeding goal is one of the most important elements of animal breeding. The breeding goal and the corresponding selection index determine the direction in which genetic improvement should take place. In all species, breeding goals have moved from primarily production-driven breeding goals to balanced breeding goals that aim for simultaneous improvement of production, efficiency, and health and functional traits (Olesen et al., 2003; Knap, 2009a; Neeteson-Van Nieuwenhoven et al., 2013; Miglior et al., 2017). Genetic improvement of resilience fits within the philosophy of balanced breeding, but, as far as we know, resilience is not (yet) included in breeding goals of livestock. If resilience has an impact on farm profit it should be in the breeding goal. In other words, if the economic value of resilience is nonzero, it should be a breeding goal trait. The question is, however, how can we determine the economic value for resilience.

To determine the economic value of resilience, we can consider the costs of a lack of resilience, for example higher production losses, (labor) costs of health treatments, veterinary costs, and labor costs of the farmer for observing animals that show signs of lack of resilience. When determining the economic value of traits, it is important to avoid double counting. If resilience is defined as fluctuations in production, care needs to be taken to avoid double counting. For example, production losses (i.e., deviations due to a lack of resilience) might already be captured by the production traits, especially in dairy cows. Furthermore, costs of health treatments might already be accounted for in the breeding goal, for instance in the case of mastitis in dairy cattle. Treatment costs of mastitis, or costs of discarded milk should not be accounted for in resilience. On the other hand, production losses and costs of diseases are not always included in the breeding goal of, for instance, pigs and poultry: there is a lack of health traits in these breeding goals, and observed production losses in commercial or crossbred environments due to disturbances may not be observed in the high health selection environment. Therefore, the economic value of resilience in pigs and poultry may include production losses in the commercial environment and health costs. Anyway, the economic value can, for example, be based on labor costs for observing animals that show signs of disease or other problems, e.g., alerts or visual signs. These costs are often overlooked, because it is considered to be part of day-to-day management. However, if the number of animals per farm employee is increasing and labor time is restricted, this is clearly associated with the farmer's requirement for healthy and easy-to-manage animals. Genetic improvement in resilience would reduce labor requirements and would allow the farmer to keep more animals. Resilience should therefore be included in breeding goals.

For sustainable animal breeding, environmental and societal concerns have to be taken into account, in addition to economic concerns (Olesen et al., 2000; Nielsen and Amer, 2007). In other words, in addition to an economic value for resilience, a non-economic value might be present. Non-economic values can require extensive work to determine (e.g., Nielsen et al., 2011; Grimsrud et al., 2013), and could for example be based on improved health and welfare of animals, and job satisfaction of farmers. However, for the sake of merely illustrating resilience as a concept, we will not take non-economic values into account in this paper.

**Figure 3:**
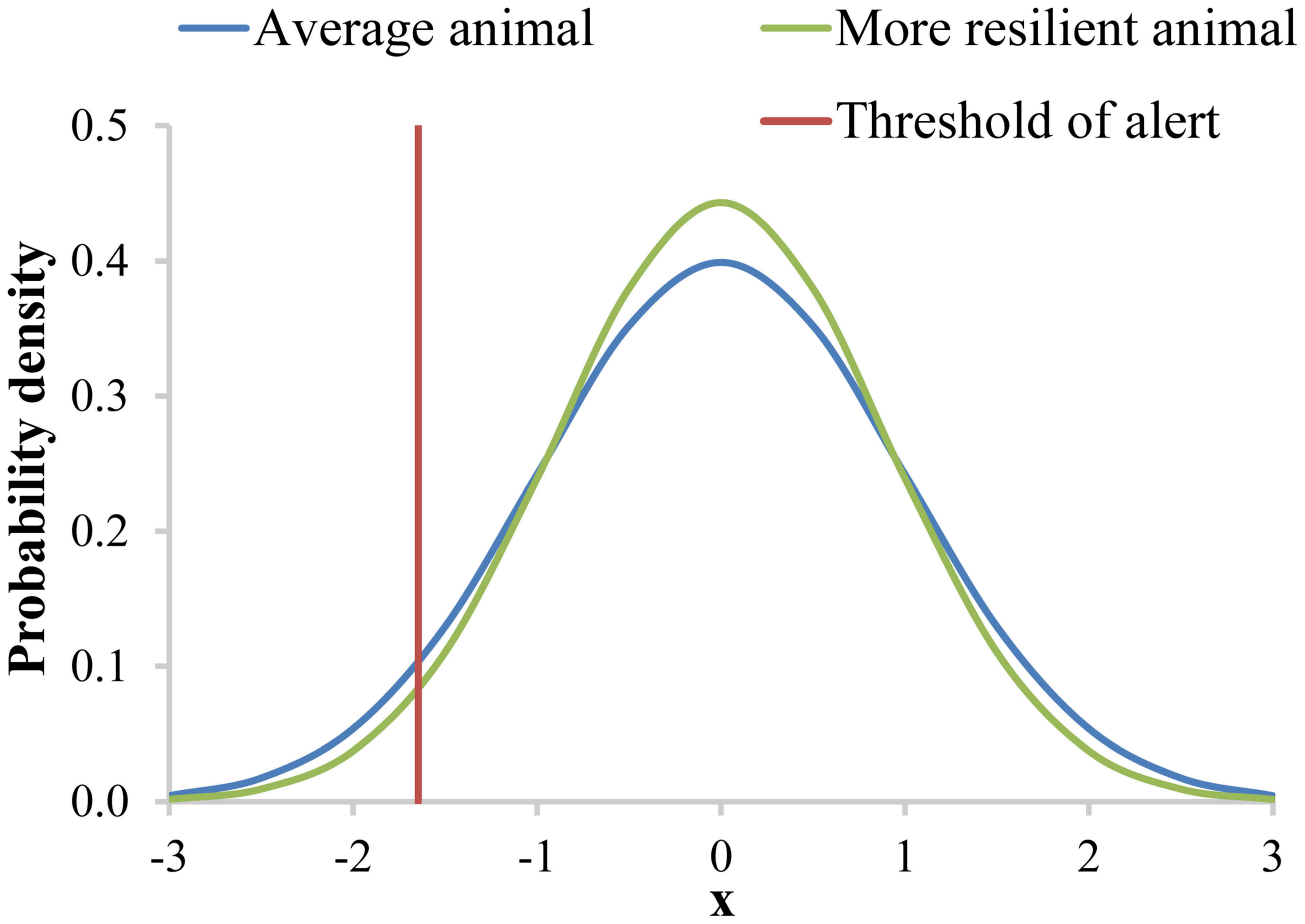
The normal distribution of the probability that an animal generates an alert with an individual-specific mean and variance based on a trait reaching a certain threshold. In this figure the individual specific variance of the “more resilient animal” is smaller than the individual specific variance of the “average animal.” Therefore, the “more resilient animal” has a lower probability to generate an alert than the “average animal”.

Next, we will use the example of labor costs to show the potential of including resilience in breeding goals. Additional labor costs for (lower) resilience are related to the probability that the animal generates an alert. An alert is a warning that might indicate that an animal is influenced by a disturbance. An alert can be generated either by visual inspection, or by sensors, AMS or AFS. We assume that alerts are generated when a trait (with a normal distribution and an individual-specific variance) exceeds a fixed threshold value that is based on the population variance (e.g., a threshold that belongs to the population-wide 1% probability). This trait could be for instance milk yield or body weight, but likely not the previously proposed resilience indicators. The proposed resilience indicators are expected to detect problems too late, and we propose to use them to breed for more resilient animals rather than to use them as predictors of alerts. Breeding for more resilient animals will result in offspring with a smaller variance in their sensor values than the current generation, and thus a smaller probability of generating an alert (Figure 3).

We will illustrate the example of using labor costs to derive economic values in two situations: in case labor time is unrestricted, and, more realistically, in case labor time is restricted per farm (i.e., maximal number of animals given farm conditions). In addition, we will show how a combined economic value for resilience can be determined based on multiple components, in this case simplified to labor costs and (health) treatment costs. For simplicity, we choose variance as the resilience indicator for (further) illustration. We also assume that breeding for a lower variance will decrease the probability of generating alerts, and that the genetic correlation between the variance and resilience in the breeding goal is 1. We hypothesize that breeding for more resilient animals will result in offspring with an autocorrelation closer to zero, a skewness closer to zero, and a slope closer to zero. In all cases, the offspring are expected to have a lower probability of generating an alert, because the underlying concept is the same, although it is less obvious than with a smaller variance.

### Economic Value for Resilience Based on one Component

If labor time is unrestricted, the economic value of resilience is the change in expected number of alerts multiplied by the time per alert and the labor cost per time unit over the whole production cycle of an individual; i.e., using Equation 8b from Mulder et al. (2008), the economic value of resilience (*v*_*resilience, unrestricted labor time*_) is:

(1) $$\begin{array}{r} v_{resilience,unrestricted\;labor\;time}=0.5\times z\times x\times l_{a}\times c_{l}\times d \end{array}$$

where *z* is the ordinate of the standard normal distribution at the standardized threshold *x* of the alert (e.g., the threshold that belongs to the 1% probability), *l*_*a*_ is the labor time required for dealing with the alert, *c*_*l*_ is the labor cost per time unit, and *d* is the number of days of the finisher period.

If labor time is restricted per farm (i.e., the number of animals on a farm is maximized given a certain farm management), we assume that the total available time (*L*) is constant, and average time available per animal for normal management (*l*_*n*_) cannot be changed. We also assume that the average time per animal required for dealing with alerts (*l*_*r*_ = *l*_*a*_×*p*_*a*_, with *p*_*a*_ being the probability of obtaining an alert) can be changed by selection, i.e., selection for resilience reduces the number of alerts per animal over the whole production cycle. The total profit of a farm is:

(2) $$\begin{array}{r} Profit=n\times \left(Revenues-Costs\right) \end{array}$$

where *n* is the number of animals per farm, equal to:

(3) $$\begin{array}{r} n=\frac{L}{l_{n}+l_{r}} \end{array}$$

Rewriting Equation (2) using Equation (3) results in:

(4) $$\begin{array}{r} Profit=\frac{L}{l_{n}+l_{r}}\times \left(Revenues-Costs\right) \end{array}$$

To obtain the economic value, the derivative of Equation 4 with respect to *l*_*r*_ is required, being:

(5) $$\begin{array}{r} \frac{dProfit}{dl_{r}}=\frac{-L}{{\left(l_{n}+l_{r}\right)}^{2}}\times \left(Revenues-Costs\right) \end{array}$$

The economic value must be expressed per animal and therefore Equation 5 is divided by Equation 3 to obtain the improvement in profit when *l*_*r*_ changes with 1 time unit:

(6) $$\begin{array}{r} \frac{dProfit}{dl_{r}/animal}=\frac{-\left(Revenues-Costs\right)}{l_{n}+l_{r}} \end{array}$$

In other words, Equation (6) is the change in profit when changing *l*_*r*_ with 1 time unit, and can be interpreted as the cost-price of labor spent on dealing with alerts for the total period an animal is kept, i.e., the product *c*_*l*_×*d* in Equation (1). In fact, the economic value shows the increase or decrease in farm profit due to higher or lower resilience, because more or fewer animals can be kept on the farm, if labor is restricted.

To obtain the economic value on the basis of a difference in resilience, Equation 1 (unrestricted labor time) is adjusted based on the different cost-price of restricted labor time of Equation 6. This results in:

(7) $$\begin{array}{r} v_{resilience,restricted\;labor\;time}=\\ \;0.5\times z\times x\times l_{a}\times \frac{-\left(Revenues-Costs\right)}{l_{n}+l_{r}} \end{array}$$

**Figure 4:**
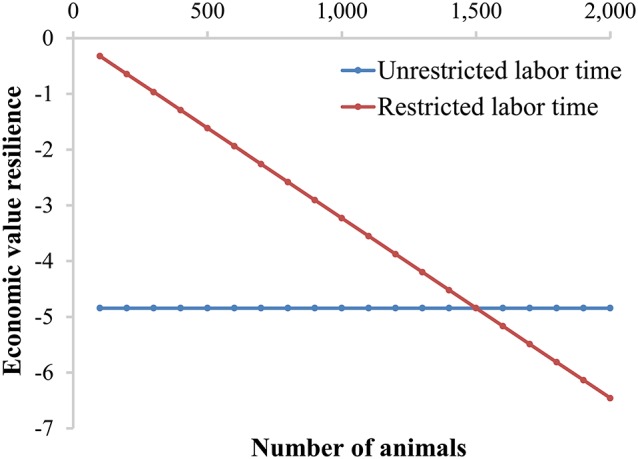
Economic value for resilience based on available labor time, either unrestricted or restricted, for day-to-day animal care taking. Assumptions made are: 8 labor h/day, 15 currency units/h labor costs, 10 currency unit profit per animal (i.e., the economic value of growth), a 1% alert probability (i.e., *x* = −2.33), 5 min attention time per alert (*l*_*a*_), and 125 days (*d*) to grow from 25 kg to 125 kg (i.e., average daily gain is 800 g/day).

To show the impact of unrestricted labor time and restricted labor time per farm, we calculate the economic values for resilience for a farm with finisher pigs. We assume 8 labor h/day, 15 currency units/h labor costs, 10 currency unit profit per animal (i.e., the economic value of growth), a 1% alert probability (i.e., *x* = −2.33), 5 min attention time per alert (*l*_*a*_), and 125 days (*d*) to grow from 25 kg to 125 kg (i.e., average daily gain is 800 g/day). Figure 4 shows that the economic value of resilience is constant when labor time is unrestricted. However, the economic value of resilience increases with increasing farm size when labor time is restricted per farm (Figure 4). The two situations lead to an equal economic value when the farm size is 1,500 finisher pigs, because in that case the income of the farmer would be 15 currency units/h, equal to the price of unrestricted labor. The economic value of resilience based on restricted labor time reaches more than 60% of the economic value of growth (in our example) with a farm size of 2,000 pigs, and would keep increasing with increasing farm size. Improving resilience of animals would thus allow more animals per farm (i.e., intensification). In fact with restricted labor time, the time for normal management (*l*_*n*_) per animal decreases with an increase in number of animals and a constant amount of labor time available. Therefore, the proportion of time for alerts (*l*_*r*_) increases and the proportion of time for normal time (*l*_*n*_) decreases with farm size. As a consequence, an increase or decrease in resilience has a larger impact on profit of the farm when the farm size increases. Nevertheless, both situations show that the economic value of resilience would be negative, meaning that reducing deviations will have a beneficial effect (i.e., an increase) on farm profit and thus resilience should be included in the breeding goal.

### Economic Value for Resilience Based on Multiple Components

In the situation above, we considered only labor costs. Next, we extend the economic value of resilience to include for instance treatment costs for a disease, in case these costs are not yet included in the breeding goal. The economic value for resilience is:

(8) $$\begin{array}{r} v_{resilience}=v_{labor}+v_{treatment} \end{array}$$

with *v*_*labor*_ being the economic value for labor costs (based either on unrestricted or restricted labor time) and *v*_*treatments*_ being the economic value for treatment costs, which can be defined as:

(9) $$\begin{array}{r} v_{treatment}=0.5\times z\times x\times p_{treatment}\times Cost_{treatment} \end{array}$$

where *z* is the ordinate of the standard normal distribution at the standardized threshold *x* of the alert, (e.g., the threshold that belongs to the 1% probability), *p*_*treatment*_ is the probability of a treatment given that the animal got an alert, and *Cost*_*treatment*_ is the cost of the treatments. Note that in this case, treatment costs must not be part of other components of the selection index to avoid double counting of these costs. This small extended example shows that different components, based on costs, can be relatively easily included in the economic value of resilience. As shown in Figure 4, the economic value of resilience can easily reach 60% of the economic value of growth, but these extra components can make the economic value even larger than the economic value of growth.

## The Added Value of Resilience In Breeding Programs

We will now show the added value of estimated breeding values for resilience to breed healthy and easy-to-manage animals in two livestock species: (1) a pig scenario, and (2) a dairy cattle scenario. In both cases, the selection indices will be simplified in order to draw general conclusions. Selection is based on truncation selection on the index (I) to maximize response in the breeding goal (H). A simplified genomics scheme is simulated. Calculation of the responses to selection were done in SelAction v2.1 (Rutten et al., 2002), using the principle of Dekkers (2007) to include genomic information.

### Pig Scenario

In this example, we describe a simplified pig scenario, in which individuals are only selected on growth rate and the breeding goal is extended with resilience. We explore the effect of adding a resilience indicator (e.g., variance of deviations) to the selection index and assume a genetic correlation of 1 between the resilience indicator and resilience in the breeding goal. Assumptions made are:

- Resilience has a heritability of 0.15 (slightly more conservative than Putz et al., 2018). The genetic correlation of resilience and growth rate is unknown and will be set to 0.25 or −0.25. A positive genetic correlation indicates that a lower resilience indicator is correlated to a lower growth rate and *vice versa* for a negative correlation. In other words, a positive correlation is unfavorable and a negative correlation is favorable for simultaneous improvement of both traits.
- Selection is done at sexual maturation. Selection is based on all available information at that moment: own performance, BLUP, full and half sibs, and genomic breeding values.
- Genomic breeding values have an accuracy of ~0.77, based on a reference population of 10,000 animals and the number of independent chromosomal segments being 1,000 (Daetwyler et al., 2008).
- The economic value of growth rate is set to 1 and −1 and the economic value of resilience will be varied between −1,000 and 1,000 to obtain an ellipse of selection responses. Note that the interesting part of the ellipse is where the response in growth rate is positive and the response in the variance is negative (i.e., improved resilience).

More information about input can be found in Supplementary Material.

**Figure 5:**
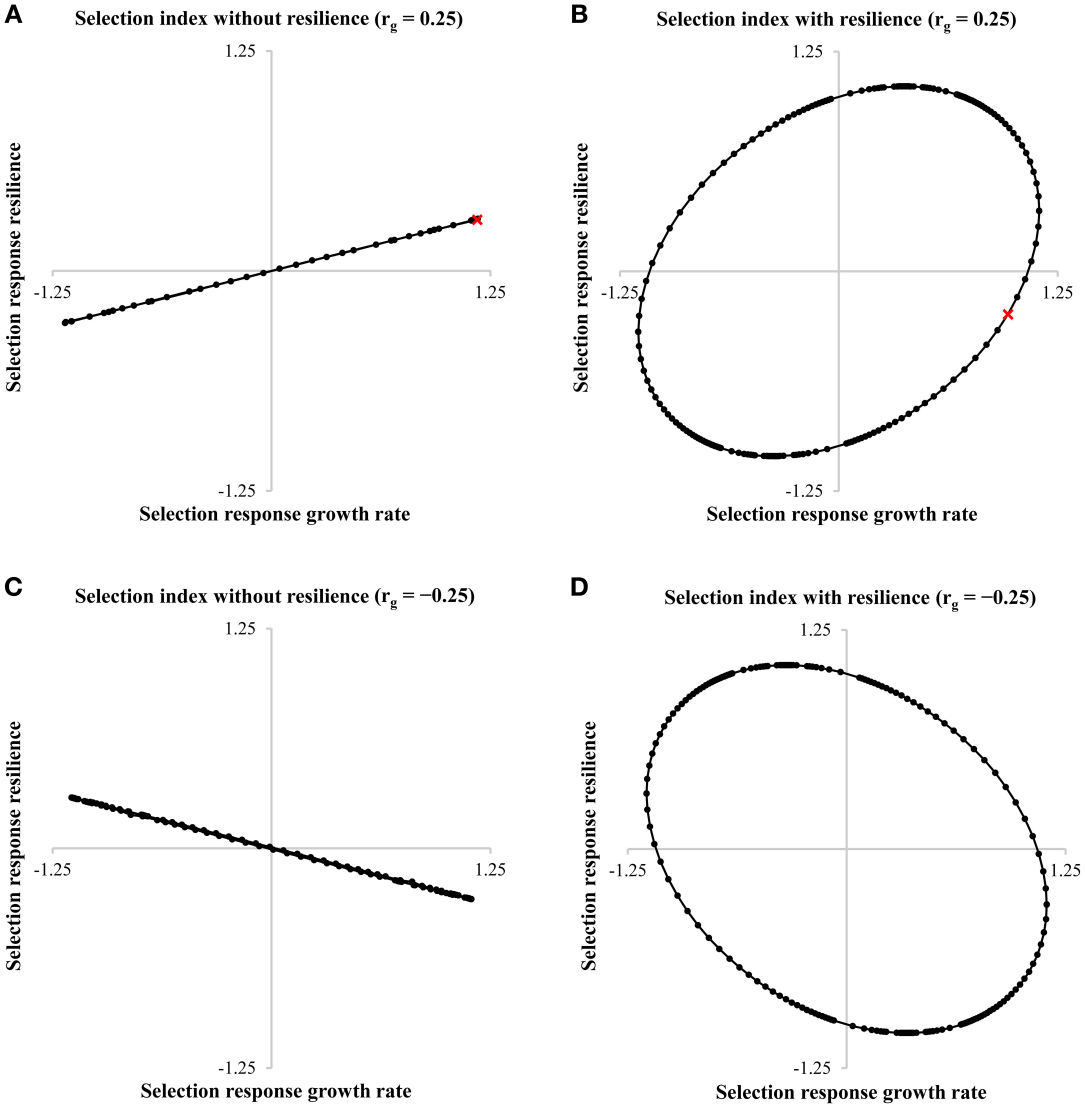
Selection responses for growth rate and resilience with the economic value of growth rate set to 1 and −1, and the economic value of resilience varying between −1,000 and 1,000 in a pig breeding program. The figure shows the response ellipses of growth rate and resilience for the selection index without resilience **(A,C)** or with resilience **(B,D)** with a genetic correlation of 0.25 **(A,B)** or a genetic correlation of −0.25 **(C,D)**. The red crosses in **A,B** are discussed in the text.

Figure 5 shows the selection response of growth rate and resilience. The desired direction of a breeding program aimed at simultaneously improving growth rate and resilience, i.e., reducing the variance, is the bottom right corner of each graph. Thus, in case of an unfavorable positive genetic correlation between growth rate and resilience, and resilience indicators are not included in the selection index, no progress can be made to obtain more resilient animals (Figure 5A). However, including a resilience indicator into the selection index of pigs can result in a higher selection response in the breeding goal (H) and more resilient animals, depending on the chosen economic values (Figure 5B). For example with an economic value of −0.6 for resilience (and an economic value of 1 for growth rate), the selection response in H is improved with 14.6% when a resilience indicator is included in the selection index. Although in this case, a reduction in the selection response of growth rate is observed (−15.4%), the selection response of resilience improves with 185.3% (see red crosses in Figures 5A,B). Not including a resilience indicator in the selection index increases the probability to generate an alert to 1.16% (start: 1%), while including a resilience indicator in the selection index reduces the probability to generate an alert to 0.88%. This corresponds to a reduction of 24.7% in the number of alerts. In case of a favorable genetic correlation between growth rate and resilience, the increase in selection response in H is even higher when comparing a selection index without (Figure 5C) and with a resilience indicator (Figure 5D): +42.8% for an economic value of −1.6 for resilience. This simplified example shows that including resilience indicators in the selection index can have big impact on the selection response and number of alerts.

### Dairy Cattle Scenario

In this example, we describe a simplified dairy cattle scenario, though with a more complex breeding program than the pig scenario: individuals are selected on milk yield and health-related traits, which are in this case longevity and udder health. We explore the effect of adding a resilience indicator (e.g., variance of deviations) to the selection index, i.e., selecting on a resilience indicator. Assumptions made are:

- Resilience has a heritability of 0.10 (Elgersma et al., 2018). The genetic correlation between resilience and milk yield is 0.61, between resilience and longevity is −0.30, and between resilience and udder health is −0.36. In other words, a higher resilience (i.e., a lower variance of deviations) is genetically correlated with a lower milk yield, a higher longevity, and a better udder health. These estimates were obtained from Elgersma et al. (2018) and CRV (2015).
- Selection is based on genomic breeding values only. Genomic breeding values have an accuracy of ~0.79, based on a reference population of 20,000 animals and the number of independent chromosomal segments being 1,200 (Daetwyler et al., 2008).
- The economic values are set to 0.3 for milk yield, 0.3 for longevity, and 0.2 for udder health. In the default situation, the economic value of resilience is set to −0.2. This means that 30% emphasis is placed on milk yield, 30% on longevity, 20% on udder health and 20% on resilience, when ignoring correlations between traits (e.g., Miglior et al., 2005). The economic value of resilience will be varied between −0.5 and −0.001 (i.e., aiming for reduced variation in deviations) as a sensitivity analysis.

More information about input can be found in Supplementary Material.

**Figure 6:**
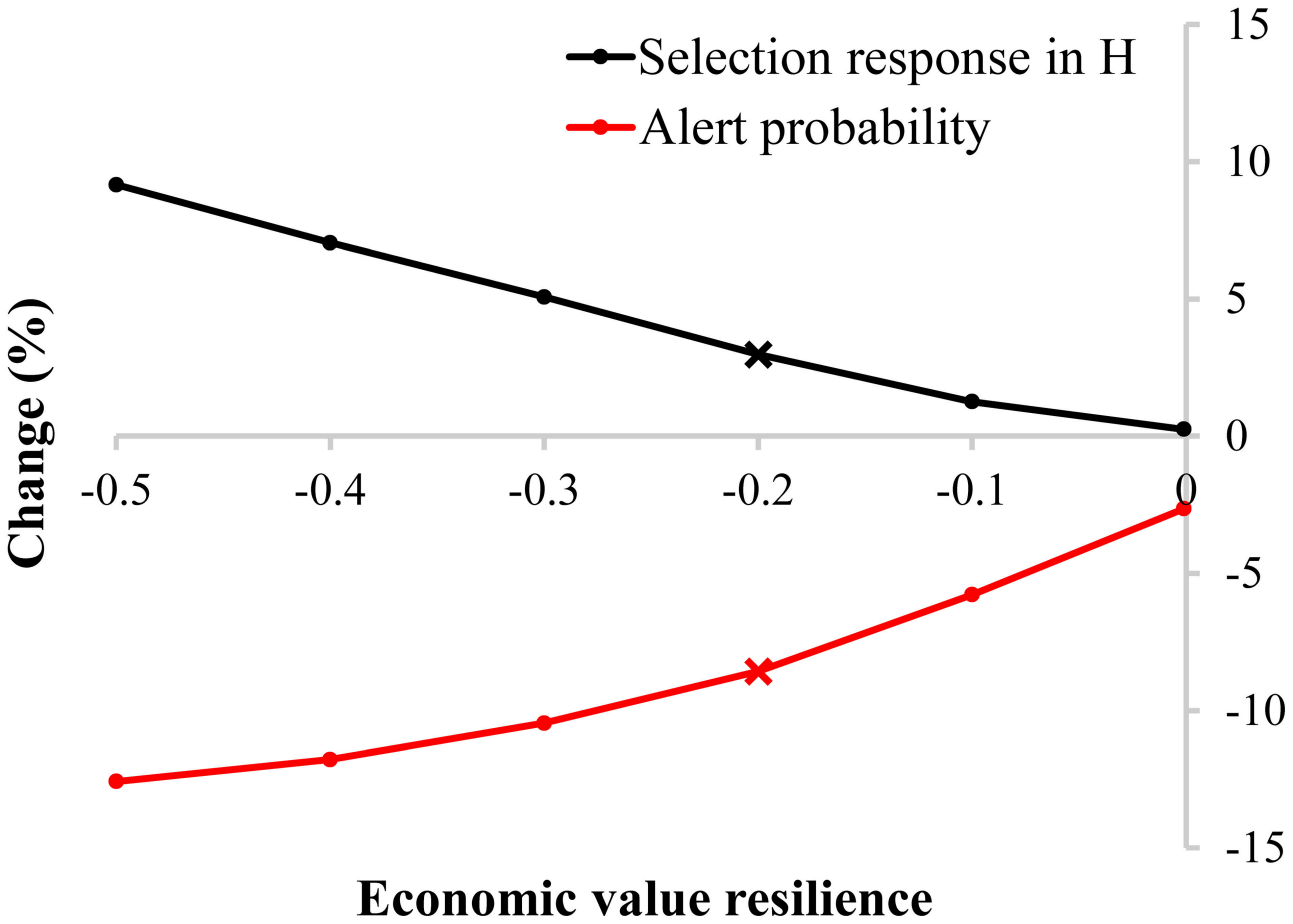
Change (in %) in selection response in the breeding goal (H) and the probability an animal generates an alert in a default selection index without and with inclusion of a resilience indicator for various economic values in a dairy cattle breeding program. The default selection index contains milk yield, longevity, and udder health. The crosses are discussed in the text and shown in detail in Table 2.

Similar to the pig scenario, including resilience into the breeding goal of dairy cattle can result in a higher selection response in H compared to not including resilience into the breeding goal, depending on the chosen economic values (Figure 6), even though health-related traits (i.e., longevity and udder health) are already included in the breeding goal and selection index. If resilience, being the variance in this case, has an economic value of −0.2, the selection response in H increases with 3.0% (see black cross in Figure 6): including a resilience indicator in the selection index compensates the loss in milk yield (6.3%) by an improvement in longevity (1.4%), udder health (1.0%), and resilience (−102.6%) (Table 2). Not including a resilience indicator in the selection index reduces the probability to generate an alert to 0.92% (start: 1%), while including a resilience indicator in the selection index reduces the probability to generate an alert to 0.84%. This corresponds to a reduction of 8.4% in the number of alerts (see red cross in Figure 6). This simplified example shows that resilience indicators can have beneficial impact. However, the effect is smaller in the dairy cattle scenario compared to the pig scenario, because of the presence of health-related traits in the selection index.

**Table 2:** Selection responses (in trait units) and alert probability (%) and their relative changes (in %) in a selection index without and with inclusion of a resilience indicator (economic value = −0.2) in a dairy cattle breeding program.

	**Trait**	**Selection index**		**Change (%)**
		**Without resilience**	**With resilience**	
Selection response	Milk yield	0.80	0.75	−6.3
(trait units)	Longevity	1.25	1.27	1.4
	Udder health	0.80	0.80	1.0
	Resilience^a^	−0.15	−0.31	−102.6
	Breeding goal (H)	0.80	0.83	3.0
	Alert probability	0.92%	0.84%	−8.4

*The default selection index contains milk yield, longevity, and udder health. Selection responses are shown for milk yield, longevity, udder health, resilience, and the breeding goal (H). ^a^In order to improve resilience of an animal, the resilience indicator trait (e.g., variation in deviations) has to be reduced. Negative selection response are therefore desired, and more negative values indicate higher resilience*.

### Perspectives and Other Livestock Species

The potential of resilience in breeding goals was clearly illustrated in the two scenarios. Obviously these scenarios overestimate the impact of resilience indicators, because of their simplification. Nevertheless, the underlying idea holds, because a reduction of time spent on an animal with an alert (for any reason) will reduce costs and consequentially increase farm profit. The pig scenario was based on only two traits, but might still be fairly close to a pig sire line scenario. Sire line breeding programs are primarily focused on improvement of production traits, in contrast to dam line breeding programs, which are more focused on improvement of reproduction and maternal traits. Nevertheless, also for dam lines resilience can be included in breeding programs: multiplier farms require resilient sows and resilient piglets. For instance, litter size uniformity has a beneficial effect on piglet resilience based on survival (Damgaard et al., 2003; Mulder et al., 2015b). Favorable correlations were found between the residual variance of feed intake and feed duration with mortality and the number of health treatments in pigs in a challenge environment (Putz et al., 2018). This shows that the residual variance of feed intake and feed duration can be used to improve resilience. Dairy cattle breeding programs contain health(-related) traits, which are expected to partly cover resilience indicators. Indeed, several favorable r_g_ between health traits and variance in deviations of milk yield were found, but none of them is equal to 1 indicating that the raw variance of milk yield contains new information about resilience and health (Elgersma et al., 2018). This was also shown in the dairy cattle scenario, which showed considerable improvement in response to a selection index with resilience. We propose that inclusion of resilience to selection indices of livestock breeding programs (similar to pigs and dairy cattle) will strongly increase the response in resilience of livestock, and likely increase the selection response in H as well.

We did not investigate resilience in breeding programs of other livestock, such as poultry, extensively kept livestock species (e.g., beef cattle and sheep), or aquaculture species, because resilience is more difficult to assess and apply in practice at this moment. In the first place, because repeated measurements are difficult to collect. This is mainly due to the impossibility to measure animals individually, due to group housing or difficulties catching individuals. In the second place, labor time spent on alerts is less relevant. Alerts are created for some livestock species based on group measurements (e.g., water intake), but not at the individual level. Also these type of alerts represent epidemic disturbances, rather than day-to-day endemic disturbances. However, resilience indicators based on a relatively limited set of production data (both frequency of repeated observations and number of animals) can already provide valuable information on health of animals (e.g., 4-weekly body weight deviations; Berghof et al., in preparation), which is currently not incorporated into the selection indices. More importantly, the development of new techniques in the near future will allow collection of daily observations on an individual level, such as individual laying nests, individual measurements of fish (without capturing), automatic collection of data, and individual tracking and measurements with camera or drones. This might result in collection of big data and the definition of new phenotypes, and can eventually result in the use of resilience indicators in breeding programs for all livestock species.

## Conclusion

This paper shows that including resilience in breeding programs has great potential to obtain healthy and easy-to-manage livestock. Resilience indicators can be based on deviations between observed production and expected production. Of particular interest are variance of deviations, autocorrelation of deviations, and skewness of deviations. Also the slope of the reaction norm might contain information, though limited to macro-environmental disturbances. An economic value for resilience indicators in the selection index can be determined based on reduced labor costs and health costs, provided that these costs are not accounted for in other traits in the selection indices. For most farms, where labor time is restricted, the economic value of resilience increases with an increasing number of animals per farm. This paper also shows the additional benefit of including resilience in the breeding goal: in both the pig and dairy cattle scenarios, we show improvements in the selection response in the breeding goals and in particular the improvement of resilience by including resilience in the breeding goal. The rapid technological development on massive collection of data (i.e., big data) is only expected to increase in the near future, resulting in more data available. The accompanying possibilities to utilize these data to determine resilience indicators, will greatly facilitate breeding for improved resilience in all livestock species.

## Author Contributions

TB and HM conceived the project. TB, MP, and HM developed the ideas about measuring resilience. HM derived the economic value of resilience, and set up the breeding scenarios. TB performed the simulations in SelAction. TB wrote the (first draft of) the paper. MP and HM contributed to writing the paper.

### Conflict of Interest Statement

This research was partly financed by the Breed4Food partners Cobb Europe, CRV, Hendrix Genetics, and Topigs Norsvin. Except for the financial contribution, no other shared interests (e.g., employment, consultancy, patents, products) exist between the Breed4Food partners and the authors. This paper was neither discussed nor reviewed by any of the Breed4Food partners. The authors declare that the research was conducted in the absence of any commercial or financial relationships that could be construed as a potential conflict of interest.
