# Supplementary material for: Opportunities to Improve Resilience in Animal Breeding Programs
Source: Front Genet. 2019 Jan 14;9:692. doi: 10.3389/fgene.2018.00692 (PMC6339870; doi:10.3389/fgene.2018.00692)
Supplement: Supplementary file 1 [file Data_Sheet_1.pdf]

Supplementary Material

Opportunities to Improve Resilience in Animal Breeding Programs

T.V.L. Berghof*, M. Poppe, H.A. Mulder

*** Correspondence:** T.V.L. Berghof: tom.berghof@wur.nl

1 Supplementary Information

The Supplementary Information describes the SelAction input used for the “4.1 Pig scenario” and “4.2 Dairy cattle scenario”.

1.1 SelAction input "4.1 Pig scenario"

- Discrete Generations: 1 - stage selection

- Traits

Number of traits: 4

Name	Var (P)	h^2	c^2	H	EV
growth	3.33	0.3	0	✓	−1 *or* 1
resilience	6.66	0.15	0	✓	*varied between* −1,000 *and* 1,000
GEBV growth	1	0.99	0	☐	0
GEBV resilience	1	0.99	0	☐	0

- Population

Number of selected parents:	50 Male	200 Female
Number of offspring per dam:	5 Male	5 Female
Proportion selected parents:	0.05 Male	0.2 Female

- Groups

1 Full-sib groups:	9 Full sibs	
1 Half-sib groups:	3 Dams	30 Half-sibs
0 Progeny groups:		

- Index

Indexes: Use identical indexes for sires and dams

**Traits**	**Index without resilience**	**Index with resilience**
growth	OP, BLUP, 9.0 FS,<br>30.0 HS of 3.0 dams	OP, BLUP, 9.0 FS,<br>30.0 HS of 3.0 dams
resilience	-	OP, BLUP, 9.0 FS,<br>30.0 HS of 3.0 dams
GEBV growth	OP	OP
GEBV resilience	-	OP

- Correlations

Phenotypic correlations are above the diagonal, genetic correlations are below the diagonal. The genetic correlation (r_g) of resilience and growth is unknown and will be set to −0.25 or 0.25. The corresponding correlations are shown as −0.25/0.25.

**r_g = −0.25/0.25**	**growth**	**resilience**	**GEBV growth**	**GEBV resilience**
**growth**	-	−0.200/−0.200	0.470/0.470	−0.110/0.110
**resilience**	−0.250/0.250	-	−0.084/0.084	0.300/0.300
**GEBV growth**	0.870/0.870	−0.220/0.220	-	−0.170/0.170
**GEBV resilience**	−0.190/0.190	0.780/0.780	−0.170/0.170	-

Common environmental correlations are all set to 0 (zero).

1.2 SelAction input "4.2 Dairy cattle scenario"

- Discrete Generations: 1 - stage selection

- Traits

Number of traits: 8

Heritabilities were obtained from Elgersma et al. (2018) and CRV (2015). Phenotypic variance were calculated such that the genetic variance was one, so that economic values are per genetic standard deviation and selection responses are in genetic standard deviations.

Name	Var (P)	h^2	c^2	H	EV
milk production	1.96	0.51	0	✓	0.3
longevity	7.14	0.14	0	✓	0.3
udder health	11.23	0.089	0	✓	0.2
resilience	10	0.1	0	✓	*varied between* −0.5 *and* −0.001
GEBV milk production	1	0.99	0	☐	0
GEBV longevity	1	0.99	0	☐	0
GEBV udder health	1	0.99	0	☐	0
GEBV resilience	1	0.99	0	☐	0

- Population

Number of selected parents:	50 Male	500 Female
Number of offspring per dam:	5 Male	5 Female
Proportion selected parents:	0.02 Male	0.08 Female

- Groups

1 Full-sib groups: 4 Full sibs

1 Half-sib groups: 9 Dams 45 Half-sibs

0 Progeny groups:

- Index

Indexes: Use identical indexes for sires and dams

**Traits**	**Index without resilience**	**Index with resilience**
milk production	-	-
longevity	-	-
udder health	-	-
resilience	-	-
GEBV milk production	OP	OP
GEBV longevity	OP	OP
GEBV udder health	OP	OP
GEBV resilience	-	OP

- Correlations

Phenotypic correlations are above the diagonal, genetic correlations are below the diagonal. Genetic correlations were obtained from Elgersma et al. (2018) and CRV (2015). Phenotypic correlations were set equal to the genetic correlations, if no information was available.

	**milk production**	**longevity**	**udder health**	**resilience**	**GEBV milk production**	**GEBV longevity**	**GEBV udder health**	**GEBV resilience**
**milk production**	-	0.370	−0.030	0.610	0.680	0.220	−0.017	0.340
**longevity**	0.370	-	0.360	−0.300	0.130	0.310	0.100	−0.089
**udder health**	−0.030	0.360	-	−0.360	−0.008	0.090	0.230	−0.085
**resilience**	0.610	−0.300	−0.360	-	0.180	−0.079	−0.088	0.250
**GEBV milk production**	0.950	0.350	−0.030	0.580	-	0.290	−0.022	−0.460
**GEBV longevity**	0.310	0.840	0.300	−0.250	0.290	-	0.230	−0.200
**GEBV udder health**	−0.023	0.280	0.770	−0.280	−0.022	0.230	-	−0.220
**GEBV resilience**	0.480	−0.240	−0.280	0.790	0.460	−0.200	−0.220	-

Common environmental correlations are all set to 0 (zero).

1.3 References

CRV (2015). "Statistical Indicators, E-20, NVI". (https://www.crv4all-international.com/wp-content/uploads/2016/03/E-20-NVI.pdf)

Elgersma, G.G., De Jong, G., Van Der Linde, R., and Mulder, H.A. (2018). Fluctuations in milk yield are heritable and can be used as a resilience indicator to breed healthy cows. J. Dairy. Sci. 101, 1240-1250. doi: 10.3168/jds.2017-13270
